# Episodic and Binge Gambling: An Exploration and Preliminary Quantitative Study

**DOI:** 10.1007/s10899-017-9697-z

**Published:** 2017-06-03

**Authors:** S. Cowlishaw, E. Nespoli, J. K. Jebadurai, N. Smith, H. Bowden-Jones

**Affiliations:** 1 School of Social and Community Medicine, University of Bristol, Bristol, BS8 2PS UK; 2 Department of Medicine and Surgery, University of Milano-Bicocca, Milan, Italy; 3 University Hospital of Wales, Cardiff and Vale University Health Board, Cardiff, UK; 4 National Problem Gambling Clinic, Central North West London NHS Foundation Trust, London, UK; 5 Department of Medicine, Imperial College London, London, UK

**Keywords:** Episodic, Binge, Gambling, Abstinence, Measurement, Treatment clinic

## Abstract

The DSM-5 includes provisions for episodic forms of gambling disorder, with such changes aligned with earlier accounts of potential binge gambling behaviours. However, there is little research that indicates the utility of these classifications of episodic or binge gambling, and this study considered their characteristics in a clinical sample. It involved administration of a new binge gambling screening tool, along with routine measures, to *n* = 214 patients entering a specialist treatment clinic for gambling problems. Results indicated that episodic gambling was common in this clinical context, with 28 and 32% of patients reporting gambling episodes that were (a) regular and alternating, and (b) irregular and intermittent, respectively. These patterns were distinguished by factors including associations with covariates that indicated differences from continuous gamblers. For example, the irregular episodic gamblers, but not the regular pattern, demonstrated lower levels of problem gambling severity and comorbidity. Rates of potential binge gambling, which was defined in terms of additional criteria, were around 4% and numbers were insufficient for comparable analyses. The findings support inclusion of episodic forms of gambling disorder in the DSM-5, but highlight the need for improved recognition and research on heterogeneous forms of episodic gambling.

## Introduction

The terms ‘pathological gambling’ or ‘gambling disorder’ describe psychiatric conditions in the ICD-10 (World Health Organization 1992) and DSM-5 (American Psychiatric Association 2013), respectively, which are characterised by persistent and recurrent maladaptive gambling that precedes gambling-related harms (e.g., severe debt, relationship breakdown). In the context of such formal diagnostic systems, the clinical entity that corresponds with maladaptive gambling has been conceptualized in various ways over time. The DSM-III (American Psychiatric Association 1980), for example, was first to incorporate a relevant diagnosis, and defined this by losses of control and disruptions to personal, family or vocational pursuits. The subsequent DSM-III-R (American Psychiatric Association 1987) included revised criteria that were modelled on substance-related disorders (e.g., the criterion for gambling with increasing amounts to achieve excitement was based on notions of tolerance) (Lesieur 1988). Notwithstanding, the diagnosis was placed in a broad category of Impulse Control Disorders. More recently, the DSM-5 (American Psychiatric Association 2013) has re-classified the corresponding diagnosis within a revised category of Substance-Related and Addictive Disorders (Petry et al. 2014), and included new provisions for specification of persistent versus episodic forms of gambling disorder. The latter reflects symptoms that subside between gambling episodes, and marks divergence from earlier versions of the DSM (Lesieur and Custer 1984). These emphasised the progressive nature of disorders, which were presumed to follow a course that commenced with gambling wins and developed through stages involving chasing losses, accumulating debts, and eventual experiences of crisis.

The new provisions for episodic forms of gambling disorder are supported by recent studies using prospective designs (Williams et al. 2015), which indicate that intermittent periods of problem gambling are common in community settings, when considered across annual intervals. The provisions are also aligned with earlier descriptive accounts of potential ‘binge gambling’ behaviours. These were outlined initially by Nower and Blaszczynski (2003), who predicated this notion on literature about bingeing behaviours that relate to consumptions of alcohol (i.e., binge drinking) and food (i.e., binge eating), as well as clinical case studies. On this basis, they proposed preliminary criteria for binge gambling, which included general characteristics of problem gambling that referenced (a) excessive expenditures relative to income, and (b) marked personal or interpersonal distress. These were additional to criteria that uniquely identified binge gamblers, and referred to (c) irregular and intermittent periods of gambling, which were characterised by (d) sudden onset, and (e) impaired control. Finally, outside of episodes, the criteria required (f) the absence of rumination, preoccupation or cravings to resume gambling. Nower and Blaszczynski (2003) theorised that gambling binges were often triggered by psychosocial stressors, and were not just linked to the availability of funds. The latter contrasts with relatively continuous descriptions of pathological gambling (Lesieur and Custer 1984), which accounted for levels of gambling that varied along with financial resources, but generally assumed that preoccupation and urges were unremitting.

Subsequent to Nower and Blaszczynski (2003), there has been surprisingly little research that has evaluated and elucidated the characteristics of this hypothesised form of gambling or problem gambling. Griffiths (2006) presented a case study that described an individual who fit some criteria for binge gambling, which included irregular and intermittent periods of gambling (which were separated by years, and were triggered by psychosocial stressors), and absences between episodes of discernible urges to resume gambling. Sklar, Gupta and Derevensky (2010) reported findings from a qualitative investigation of adolescents in drug treatment (*n* = 14) which also yielded descriptions of time-limited gambling episodes that were potentially consistent with binge gambling. However, the unique setting generated findings that were not applicable in alternative contexts (e.g., gambling episodes were often described as a means of acquiring funds to purchase drugs), and were not unambiguous indicators of binge gambling criteria. We are aware of no other relevant studies in the published literature, and this includes any quantitative data sources. As such, the utility of Nower and Blaszczynski’s (2003) conceptualisation of binge gambling, including the capacity of criteria to identify a meaningful sub-group of gamblers or problem gamblers, remains currently unclear.

Notwithstanding the absolute dearth of relevant evidence, there are reasons to expect that patterns of episodic or binge gambling could have important implications across community and clinical settings. It seems plausible, for example, that such patterns might indicate behaviours that are problematic but are not appropriately recognised. Rather, they may be classified as problems that fall across lower levels of the spectrum of severity, or alternatively, gambling disorders in remission. Such classifications seem likely when assessments coincide with periods of abstinence (e.g., when administered within epidemiological surveys). In contrast, evaluations conducted during gambling episodes seem more likely to identify relevant disorders. These evaluations may be expected in treatment services, where current gambling and associated problems have precipitated help-seeking. In such contexts, episodic or binge gambling may indicate the need for unique assessment strategies and recognition of gambling cessation as an ambiguous indicator of recovery.

In the context of scant empirical evidence, there remains a clear need for new research on episodic or binge gambling behaviours, which has become increasingly salient given the incorporation of episodic forms of gambling disorder in the DSM-5. The current study provides a preliminary response to this need, and involved examination of the extent and nature of episodic or binge gambling among patients accessing treatment for gambling problems. The primary objectives were to:

- Evaluate the frequency and characteristics of different types of episodic and continuous patterns of gambling in treatment for gambling problems;
- Examine the frequency of potential binge gambling, as defined by criteria from Nower and Blaszczynski (2003); and
- Evaluate whether episodic or binge gambling could be distinguished from continuous gambling in terms of clinical characteristics.

A secondary objective was to describe a new measure that may provide a standardised means of assessing patterns of episodic or binge gambling.

## Method

### Participants and Procedure

Participants comprised *n* = 214 patients who were accessing a specialist treatment service in London, and involved data collection across eleven months. This initially extended from June 2012 to January 2013, with a second period of data collection (to increase sample size) from June to September 2015. The only inclusion criteria was that patients were referred to the clinic for the first time. The resulting sample was mostly male (*n* = 191, 89.3%), and was single (*n* = 98, 45.8%), married/cohabitating (*n* = 83, 38.8%), or divorced/separated/widowed (*n* = 15, 7.0%). Participants were predominantly white (*n* = 158, 73.8%) and represented various age groups: 16–24 (*n* = 28, 13.1%), 25–34 (*n* = 74, 34.6%), 35–44 (*n* = 64, 29.9%), and +45 years (*n* = 45, 21.0%). Participants were typically employed full-time or part-time (*n* = 140, 65.4%), were unemployed and looking for work (*n* = 26, 12.1%), or were not in the labour force (*n* = 32, 15.0%). The largest minority of patients reported annual personal incomes below £20,000 (*n* = 77, 36.0%), with smaller numbers describing incomes between £20,000 and 39,999 (*n* = 37, 17.3%) or above £40,000 (*n* = 23, 10.7%). The remainder (*n* = 77, 36.0%) failed to provide data on income. According to the Problem Gambling Severity Index (PGSI) (Ferris and Wynne 2001), there was 89.7% of patients (*n* = 192) that were problem gamblers (PGSI = 8 +) at intake, with smaller numbers classified as moderate-risk (*n* = 3, 1.4%) or low-risk (*n* = 2, 0.9%) gamblers.

### Measures

*Binge Gambling Screening Tool (BGST)*: The BGST is a 9-item tool that was developed by two of the authors to provide a preliminary means of operationalising the binge gambling criteria in this clinical setting. The initial item provided a diagrammatic representation of four patterns of gambling, and asked respondents to indicate the best reflection of their gambling. Patterns corresponded to: (a) *continuous* gambling most days of every week without a break; (b) *alternating* gambling and non-gambling following regular patterns; (c) *rare* gambling episodes; and (d) *intermittent and irregular* gambling. The latter reflected periods of gambling that were variable in length and were interspersed with periods of non-gambling. Respondents who indicated patterns of intermittent or episodic gambling (b-c) were then asked eight core questions about periods of gambling (e.g., During this episode, did you feel you lost control over gambling?) and abstinence (e.g., Did you get any urge to gamble when you were not gambling). Items were scored on a binary response scale (0 = *no*, 1 = *yes*) and are shown in Appendix A.

Supplementary items in the BGST included questions about duration of the last gambling episode, and the last period of non-gambling. Responses to both items were recorded using an open response format (referring to days, weeks or months), and were re-coded to derive a 7-point indicator ranging from 0 (<24 h) to 6 (2+ years). Patients also ticked boxes indicating reasons for stopping gambling (e.g., no funds available; funds available, but no urge). There were comparable questions about reasons for re-starting gambling (e.g., uncontrollable urge; major life crisis).

Routine data were also obtained on clinical characteristics. These included measures administered through questionnaires that were completed in advance of initial consultations, and were subsequently explored or verified by clinicians. This questionnaire asked patients whether they had ever lost a relationship because of gambling, lost a job because of gambling, or had committed illegal acts to finance gambling. Responses to each item were scored using a binary response format (*no*, *yes*). There was another item about estimated debts, which asked how much money was owed, and to whom, and was answered using an open response format. For current purposes, this measure was collapsed to form a categorical variable reflecting lowest, middle and highest tertiles. Items also asked whether anyone else in the family had a problem with gambling (and if so, who), or had any other mental health problem or addiction (e.g., anxiety, depression or alcohol misuse). These were used to form indicators of parental gambling and family psychiatric history, respectively. Measures also included the PGSI (Ferris and Wynne 2001), which consists of 9-items that relate to past year experiences and are scored on a 4-point response scale (0 = *Never*, 3 = *Almost always*). Scores were collapsed according to conventional criteria that indicate low-risk (PGSI = 1–2), moderate risk (PGSI = 3–7) and problem gambling (PGSI = 8+). The 9-item Patient Health Questionnaire (PHQ) (Kroencke et al. 2001) provided a measure depression symptoms, while the GAD-7 (Spitzer et al. 2006) provided a measure of anxiety (both in the past 2-weeks).

On the basis of all information obtained through questionnaires and inquiries made during consultations (e.g., incorporating patient reports of prior diagnoses or medication usage), there was another classification of whether patients indicated any recognised psychiatric comorbidity (*yes*, *no*). Finally, there was a clinician determination made of the overall severity of client difficulties, which were classified as low, medium or high. This was formed on the basis of various factors including history of gambling and motivations for gambling (e.g., chasing rewards, avoidance of punishment), psychiatric comorbidity and treatment goals (e.g., abstinence). For current purposes, this measure was collapsed to form a variable comprising values of 0 (low or moderate severity) and 1 (high severity). Further details can be obtained from the corresponding author.

### Data Analyses

Data-file preparation and preliminary analyses were conducted using SPSS Version 21, while subsequent analyses used Program R. These initially comprised descriptive analyses of items from the BGST, which provided evaluations of the frequency of continuous and episodic gambling, with χ^2^ analyses evaluating socio-demographic differences across groups. We then produced descriptive analyses of items indicating the duration of episodes of gambling and non-gambling, reasons for stopping or re-starting, and binge gambling criteria. We evaluated some preliminary operational definitions of binge gambling, and considered implications for prevalence. Finally, we then considered associations with episodic or binge gambling and covariates. These were evaluated through logistic regression models, which specified the binary indicators of episodic or binge gambling as endogenous variables, and with clinical characteristics treated as exogenous. Covariates included measures of problem gambling severity (e.g., PGSI scores), psychosocial problems (e.g., estimated debt), comorbidities (e.g., depression scores), and family psychiatric history. Each characteristic was evaluated in a separate regression model, which thus provided estimates of bivariate association with episodic or binge gambling. Odds Ratios (ORs) with 95% confidence intervals (CIs) indicated the magnitude of associations.

## Results

We initially evaluated the frequency of continuous versus episodic patterns of gambling, as indicated by the preliminary item from the BGST. This yielded *n* = 79 patients (36.9%) who reported continuous gambling, in comparison with *n* = 68 (31.8%) indicating intermittent and irregular gambling, *n* = 59 (27.6%) describing alternating (i.e., regular) gambling, and *n* = 8 (3.5%) patients reporting rare (one off) gambling episodes. Thus, there were *n* = 127 patients (59.4%) who reported gambling that was episodic and re-occurring (excluding rare episodes). Table 1 shows the socio-demographic characteristics across the continuous, regular, and irregular episodic profiles. χ^2^ analyses indicated that groups were similar on socio-demographic characteristics except for relationship status. Relative to the continuous profile, the irregular episodic gamblers were more likely to be married or cohabitating, and were less likely to be single.

**Table 1:** Socio-demographic characteristics according to continuous, alternating (regular) episodic, and irregular and intermittent episodic gambling profiles

	Continuous [0] (*n* = 79)		Regular episodic [1] (*n* = 59)		Irregular episodic [2] (*n* = 68)		[0] versus [1]		[0] versus [2]	
							*χ* ^2^	*p*	*χ* ^2^	*p*
	*n*	*%*	*n*	*%*	*n*	*%*				
Sex (male)	68	88.3	54	93.1	62	92.5	0.41	0.522	0.33	0.568
Age (years)							0.78	0.854	3.12	0.373
16–24	13	16.9	10	17.2	5	7.4				
25–34	25	32.5	18	31.0	26	38.2				
35–44	22	28.6	20	34.5	20	29.4				
45+	17	22.1	10	17.2	17	25.0				
Race (white)	59	78.7	45	78.9	49	75.4	0.00	1.000	0.07	0.795
Relationship status							1.97	0.374	10.91	0.004
Single	41	57.7	34	61.8	19	29.7				
Married/cohabitating	27	38.0	16	29.1	39	60.9				
Separated/divorced/widowed	3	4.2	5	9.1	6	9.4				
Employment (past 12 months)							2.63	0.268	1.88	0.391
Employed	45	63.4	42	76.4	49	74.2				
Unemployed	12	16.9	5	9.1	8	12.1				
Not in the labour force	14	19.7	8	14.5	9	13.6				
Annual personal income							0.02	0.990	2.41	0.300
£0–19,999	33	61.1	22	61.1	20	47.6				
£20,000–39,999	14	25.9	9	25.0	12	28.6				
≥£35,000	7	13.0	5	13.9	10	23.8				

Table 2 shows responses to items about the duration of gambling and non-gambling episodes. When asked about the last episode of gambling, the modal response across regular and irregular episodic gamblers was 1–6 days, with more than 90% of patients reporting episodes lasting 6-months or less. The item about the duration of abstinence indicated that 1–4 weeks was the modal response. Around 90% of both groups reported abstinence lasting for 6-months or less, with approximately 10% indicating periods of 12-months or more. The only discernible difference across regular and irregular episodic profiles was that the latter were seemingly less likely to report periods of non-gambling that lasted for less than one week.

**Table 2:** Frequency analyses for duration of gambling episodes and periods of abstinence across regular (alternating) and irregular episodic gamblers

		Regular episodic		Irregular episodic	
		(*n* = 59)		(*n* = 68)	
		*n*	*%*	*n*	*%*
*Duration of last gambling episode*					
<24 h		8	15.7	5	8.3
1–6 days		25	49.0	31	51.7
1–4 weeks		10	19.6	12	20.0
1–6 months		4	7.8	8	13.3
6–12 months		1	2.0	1	1.7
1–2 years		2	3.9	1	1.7
2 + years		1	2.0	2	3.3
*Duration of last abstinent (non-gambling) episode*					
1–6 days		10	20.8	7	11.9
1–4 weeks		17	35.4	23	39.0
1–6 months		15	31.3	22	37.3
6–12 months		1	2.1	1	1.7
1–2 years		3	6.3	4	6.8
2 + years		2	4.2	2	3.4

Reasons given for stopping and re-starting gambling are provided in Table 3. As shown, it was common for both groups to report cessation of gambling because of adverse consequences, while large numbers reported stopping because of insufficient funds. It was common for both groups to describe re-starting because of an uncontrollable urge, or because funds were available. There were small numbers reporting stopping or re-starting because of life crises. χ^2^ tests indicated no significant differences in reasons for stopping or re-starting across regular and irregular episodic profiles.

**Table 3:** Frequency analyses of reasons for stopping and re-starting gambling episodes, as well as BGST items that operationalise the binge gambling criteria, compared across regular (alternating) and irregular episodic gambling profiles

	Regular episodic		Irregular episodic		*χ* ^2^	*p*
	*n* = 59		*n* = 68			
	*n*	*%*	*n*	*%*		
*Reasons for stopping gambling*						
No funds available	37	67.3	36	57.1	0.88	0.347
Funds available, but no urge	17	30.9	22	34.9	0.07	0.790
Gambling consequences (e.g. debts, job loss)	38	69.1	49	77.8	0.74	0.390
End of the crisis that caused gambling	5	9.3	8	12.7	0.09	0.768
*Reasons for re-starting gambling*						
Uncontrollable urge	39	73.6	37	59.7	1.88	0.170
Major life crises occurred	8	15.4	9	14.5	0.00	1.000
Pay day/funds available	32	60.4	38	61.3	0.00	1.000
*Binge gambling criteria*						
Gamble suddenly after a period of abstinence	32	55.2	54	80.6	8.21	0.004
Episode began with sudden uncontrollable urge	44	74.6	53	79.1	0.15	0.696
Episode precipitated by stressful event	19	33.9	24	35.8	0.00	0.977
Increases in frequency/intensity of gambling during episode	45	77.6	52	78.8	0.00	1.000
Loss of control during episode	57	96.6	61	91.0	0.83	0.362
Gambling stopped abruptly	24	41.4	50	75.8	13.77	0.000
Urges to gambling when not gambling	42	76.4	45	73.8	0.01	0.915
Pre-occupation with gambling when not gambling	30	55.6	22	34.9	4.21	0.040

Table 3 displays frequencies for BGST items which operationalised the proposed criteria for binge gambling. As shown, there were around three quarters of both groups of episodic gamblers that reported gambling which commenced with sudden urges, and around 90% reported losses of control during episodes. In reference to abstinent periods, there were around 75% reporting urges which remained present outside of episodes. In contrast, there were smaller numbers reporting preoccupation that remained present. χ^2^ analyses indicated three significant differences across regular and irregular gambling profiles. Irregular episodic gamblers were more likely to report gambling suddenly after a period of abstinence, and stopping gambling abruptly. They were less likely to report pre-occupation outside of episodes.

Analyses were then conducted to evaluate some preliminary operational definitions of binge gambling. The first was based on a strict interpretation of criteria from Nower and Blaszczynski (2003) (*operational definition a*), which identified binge gambling according to the following criteria (specific item responses shown in parentheses refer to Appendix A):

1. Gambling was irregular and intermittent (Item 1 = ‘D’);
2. Gambling episodes began suddenly after a period of abstinence (Item 3 = ‘yes’);
3. During episodes, there were losses of control over gambling (Item 7 = ‘yes’);
4. Outside of episodes, there was absence of pre-occupation with gambling (Item 10 = ‘no’);
5. Outside of episodes, there was absence of gambling urges (Item 11 = ‘no’).

According to these criteria, there were *n* = 8 patients (3.7%) that were classified as binge gamblers. Exploratory analyses then evaluated specific criteria, and indicated that many irregular gamblers were excluded by virtue of requirements for the absence of urges outside of episodes. When excluding this criterion, for example, the results indicated *n* = 29 (13.6%) patients that were potential binge gamblers (*operational definition b*).

In the final stage of analyses, we evaluated potential covariates of episodic and binge gambling through logistic regression models. These treated the continuous gamblers as the comparison group, and considered associations with (a) regular episodic gambling, (b) irregular episodic gambling, and (c) *operational definition b* for binge gambling. Comparisons with the latter were not expected to yield significant effects (given small numbers), but are presented for exploratory purposes. Small amounts of missing data were managed through pairwise deletion, and results are shown in Table 4.

**Table 4:** Frequency and logistic regression analyses of clinical covariates, comparing the alternative classification of episodic gambling with the continuous gambling profile (which was the reference category for all regression models)

	Continuous gamblers [0] (*n* = 79)		Regular episodic [1] (n = 59)		Irregular episodic [2] (n = 68)		Binge gamblers [3] (n = 29)		[0] vs [1]			[0] vs [2]			[0] vs [3]		
									OR	95% CI		OR	95% CI		OR	95% CI	
	*n*	*%*	n	%	n	%	n	%		LB	UB		LB	UB		LB	UB
*Problem gambling severity*																	
PGSI score, Mean (SD)	20.57	(4.56)	19.71	(5.68)	18.07	(5.61)	18.19	(5.57)	0.97	0.90	1.04	0.91^**^	0.84	0.97	0.91^*^	0.82	0.99
Severity rating (high)	14	18.2	11	19.3	3	4.7	2	7.4	1.08	0.44	2.58	0.22^*^	0.05	0.72	0.36	0.05	1.41
*Psychosocial problems*																	
Estimated debt (high)	27	36.5	19	33.9	16	28.1	8	36.4	0.89	0.43	1.85	0.68	0.32	1.42	0.99	0.36	2.64
Job loss	14	18.2	13	22.4	5	7.8	1	3.7	1.30	0.55	3.04	0.38^†^	0.12	1.06	0.17^†^	0.01	0.93
Relationship loss	22	28.6	24	42.1	12	18.8	5	18.5	1.82	0.89	3.77	0.58	0.25	1.27	0.57	0.17	1.59
Illegal acts (any)	40	51.9	22	37.9	23	36.5	9	34.6	0.57	0.28	1.13	0.53	0.27	1.04	0.49	0.19	1.21
*Comorbidities*																	
Any comorbidities	46	59.0	29	50.0	27	42.2	12	44.4	0.70	0.35	1.38	0.51^*^	0.26	0.99	0.56	0.23	1.34
PHQ score, Mean (SD)	13.43	(7.50)	13.81	(7.06)	11.3	(7.08)	11.85	(7.29)	1.01	0.96	1.06	0.96^†^	0.92	1.01	0.97	0.91	1.03
GAD-7 score, Mean (SD)	11.08	(6.69)	10.93	(5.85)	8.13	(6.00)	8.44	(5.97)	1.00	0.94	1.05	0.93^*^	0.88	0.98	0.94^†^	0.87	1.01
*Family history*																	
Parental gambling	24	31.2	12.0	20.7	22	34.4	8	29.6	0.58	0.25	1.26	1.16	0.57	2.35	0.93	0.34	2.37
Family psychiatric history	25	32.9	15.0	25.9	27	42.9	13	50.0	0.71	0.33	1.51	1.53	0.77	3.07	2.04	0.82	5.10

*OR* Odds ratio

NB. ^**^ = *p* < 0.01, ^*^ = *p* < 0.05, ^†^ = *p* < 0.10

As shown, there were no significant associations with the regular gambling profile and any clinical characteristic. In contrast, the irregular episodic gamblers demonstrated lower scores on the PGSI, relative to continuous gamblers, and were less likely to be rated as suffering ‘high severity’ difficulties. They were less likely to report any psychiatric comorbidities, and also reported lower anxiety according to the GAD-7. There were trends (*p* < 0.10) suggesting lower depression according to the PHQ, and lower rates of job loss. Analyses of potential binge gambling indicated generally similar patterns of association, which included potentially meaningful effects for lower PGSI scores (*p* < 0.05), anxiety scores (*p* < 0.10) and rates of job loss (*p* < 0.10).

## Discussion

This paper describes a preliminary examination of episodic or binge gambling in patients seeking treatment for gambling problems. Results indicated that episodic gambling was common in this setting, with around 60% of patients reporting gambling that was intermittent and recurring. As far as we know, this is the first study yielding data on the frequency of such behaviours in a clinical context, and supporting inclusion of episodic forms of gambling disorder in the DSM-5. It also provided descriptive evidence about the characteristics of these periods of gambling and non-gambling. For example, the results indicated that around half of episodic gamblers reported brief periods of active gambling, lasting from 1 to 6 days, with episodes longer than six months being comparably rare. Periods of abstinence usually lasted between 1 and 6 months. However, there were around 10% of episodic gamblers that described extended periods of non-gambling for 12 months or longer. Such results are aligned with prospective studies which indicate that intermittent periods of problem gambling are common (Williams et al. 2015). Notwithstanding, they also suggest that these community studies, which typically schedule assessments annually, may underestimate the extent of variability in periods of gambling and abstinence, which often cycle rapidly and across shorter periods.

The results indicated distinctions across different types of episodic gambling, with around 28% of patients indicating regular episodes, and 32% reporting irregular periods of gambling and non-gambling. These were distinguished by some self-reported attributes of episodes and periods of abstinence, as well as associations with clinical characteristics. The regular gamblers were more likely to report pre-occupation with gambling outside of episodes, when compared to the irregular profile, while there were no significant associations with the former pattern and covariates (indicating differences from continuous gambling). In contrast, the irregular episodic gamblers demonstrated lower levels of problem gambling severity (when compared to continuous gamblers), and lower levels of psychiatric comorbidity and anxiety. There were marginally significant trends suggesting lower levels of depression and fewer instances of job loss. These contrasting patterns may suggest that regular episodic gamblers are broadly similar to continuous gamblers, in that both tend to experience unremitting preoccupation and urges, but with time-limited episodes that are determined by external factors (e.g., absence of funds), or lower frequency patterns of gambling (e.g., on weekends). In contrast, the intermittent and irregular pattern may provide a closer approximation to descriptions of binge gambling (Nower and Blaszczynski 2003).

Findings of lower gambling severity and comorbidity among irregular episodic gamblers are consistent with accounts of Nower and Blaszczynski (2003), who argued that periods of non-gambling may provide opportunities for individuals to recoup losses through legitimate means, and otherwise contain the negative the consequences of gambling. However, by assuming that such episodic behaviours are less likely to precipitate acute crises (e.g., severe debt) that trigger help-seeking (Evans and Delfabbro 2005), it also stands that burdens of these patterns will be observed mainly in non-clinical settings, and among low to moderate severity gamblers. Research suggests that these ‘subclinical’ problem gamblers are an important group, and may account for up to 85% of the burden of gambling on public health (Browne et al. 2016). This is by virtue of their larger numbers, and also the interpersonal consequences of gambling problems (Cowlishaw et al. 2016), which imply evens greater numbers of people affected. Future studies in community settings are thus required, and may indicate a form of problematic consumption that is analogous to binge drinking. The latter is also excluded from criteria for alcohol use disorders, and rather, describes a pattern of misuse that is common in community settings (Courtney and Polich 2009), and has major consequences for public health (e.g., when considered relative to non-bingeing consumption patterns; Viner and Taylor 2007).

In contrast with findings of high rates of episodic behaviours generally, the results indicated that instances of binge gambling, which were defined in accordance with Nower and Blaszczynski (2003), were identified at lower levels in this setting. The study identified around 4% of patients that were binge gamblers according to an operational definition that was aligned with Nower and Blaszczynski (2003). The estimate was around 16% when requirements for absence of urges outside of episodes was relaxed. Although these levels are arguably non-trivial, the small numbers prohibited substantive tests of association with covariates. As such, the study could not evaluate whether binge gamblers were meaningfully distinguished from continuous gamblers, or the broader group of irregular episodic gamblers. Accordingly, there remains a need for additional studies on the Nower and Blaszczynski (2003) criteria, which includes research on rates and correlates in both clinical and community settings.

The BGST was developed to facilitate identification of episodic or binge gambling in a clinical context, and may provide a basis for future inquiries. If administered in non-clinical or community settings, then this scale should be considered in conjunction with general measures of problem gambling severity which can establish the degree of harm from gambling. However, this research should consider revisions to the BGST, and also the criteria for binge gambling, with such changes informed by developing literature on analogous behaviours. Binge eating, for example, is defined mainly by consumption of larger amounts of food than normal during short periods of time (with normal defined subjectively by larger amounts than most people would consume in similar circumstances). Such episodes are defining features of Binge Eating Disorder (American Psychiatric Association 2013), which also requires that behaviours occur, on average, at least once per week over three months. Definitions of binge gambling that were aligned with these approaches might thus consider excluding requirements for complete absence of urges or pre-occupation outside of episodes, which are unique to definitions of binge gambling. These could also consider criteria that specifies the frequency of episodes and durations across which behaviours must occur (e.g., past six months). The latter seem important in the context of gambling, where episodic behaviours occur for external reasons (e.g., absence of funds), and where notions of recovery are poorly understood (Nower and Blaszczynski 2008). A criterion for duration could thus differentiate binge gambling from other instances of episodic gambling which may be better classified in terms of recovery and relapse. Such conceptual developments will also allow specification of the necessary features of the binge gambling construct, as well as sensible limits, and would thus provide a platform for future evaluations and improvements to the psychometric properties of instruments like the BGST.

### Limitations

This was a preliminary study and findings should be considered in light of limitations. The sample comprised patients seeking treatment and findings have limited generalizability to non-clinical settings. There were small numbers of binge gamblers, as well as ‘rare episodic’ gamblers, and this indicates the need for larger samples from clinical populations that could inform suitable examinations of these groups. Analyses of covariates that compared across continuous and episodic gambling where characterised by small samples overall, and these provided low levels of statistical power. The study also described a preliminary application of the BGST, which was developed initially for clinical purposes, and was not considered in the context of a focussed evaluation of psychometric properties due to resource limitations. As such, the findings should be interpreted cautiously given the untested properties and limitations of this measure. The latter includes usage of retrospective recall to define continuous or episodic gambling, as well as experiences (e.g., absence of urges) that characterised periods of gambling and abstinence.

## Conclusions

This study indicated that patterns of episodic gambling were normative in patients seeking treatment for gambling problems, and supports provisions for episodic forms of gambling disorder in the DSM-5. However, the results also indicated potential heterogeneity in episodic gambling, which includes regular patterns and irregular episodic gambling. In this study, these patterns were distinguished by characteristics of gambling and non-gambling periods (e.g., absence of preoccupation), and associations with covariates that suggested differences from continuous gambling. Such findings highlight the strong need for further research on the nature and implications of episodic gambling in clinical settings, as well as non-clinical contexts, where such patterns of gambling are perhaps most likely to be encountered.

The study indicated that instances of binge gambling, which were defined in accordance with Nower and Blaszczynski (2003), could be reliably identified in this context at lower levels. The numbers were non-trivial, but were too small for meaningful tests of whether binge gamblers were distinguished from continuous gambling, or the broader group of irregular episodic gamblers. In the absence of supporting evidence, the clinical utility of the proposed criteria for binge gambling remains unclear. This indicates need for additional research on definitions of binge gambling, which should be considered in the context of literature on alternative binging behaviours. This may suggest new conceptualisations for binge gambling, or potential improvements to existing criteria (e.g., by including criteria for frequency and duration of episodes).

Notwithstanding the dearth of relevant evidence, the results of this preliminary study have immediate clinical implications. They indicate the need for collection of routine information to enhance recognition of episodic gambling disorders in treatment services. The BGST was developed for a comparable purpose, and provides information about patterns of gambling, and the duration of periods of gambling and non-gambling. As such, these items could be administered routinely during treatment intake for purposes of case conceptualisation. There is also a need for unique assessment and treatment strategies that are appropriate for episodic gamblers. In particular, the results suggest that immediate reductions in gambling behaviours are ambiguous indicators of effects of treatment for episodic gamblers, who may require long term follow-up. The study also indicates value from treatment components that are heavily focussed on avoiding the onset of new gambling episodes, and maintaining the beneficial effects of treatment via enhanced relapse prevention.

## References

[CR1] American Psychiatric Association (1980). Diagnostic and statistical manual of mental disorders.

[CR2] American Psychiatric Association. (1987). Diagnostic and statistical manual of mental disorders (3^rd^ ed., revised). Washington, DC: Author.

[CR3] American Psychiatric Association (2013). Diagnostic and statistical manual of mental disorders.

[CR4] Browne M, Langham E, Rawat V, Greer N, Li E, Rose J, Rockloff M, Donaldson P, Thorne H, Goodwin B, Bryden G, Best T (2016). Assessing gambling-related harm in Victoria: A public health perspective.

[CR5] Courtney KE, Polich J (2009). Binge drinking in young adults: Data, definitions, and determinants. Psychological Bulletin.

[CR6] Cowlishaw S, Suomi A, Rodgers B (2016). Implications of gambling problems for family and interpersonal adjustment: Results from the Quinte Longitudinal Study. Addiction.

[CR9] Evans L, Delfabbro PH (2005). Motivators for change and barriers to help-seeking in Australian problem gamblers. Journal of Gambling Studies.

[CR10] Ferris J, Wynne H (2001). The Canadian Problem Gambling Index.

[CR11] Griffiths MD (2006). A case study of binge problem gambling. International Journal of Mental Health and Addiction.

[CR12] Kroencke K, Spitzer R, Williams J (2001). The PHQ-9: Validity of a brief depression severity measure. Journal of General Internal Medicine.

[CR13] Lesieur HR (1988). Altering the DSM-III criteria for pathological gambling. Journal of Gambling Behavior.

[CR14] Lesieur HR, Custer RL (1984). Pathological gambling: Roots, phases, and treatment. The Annals of the American Academy of Political and Social Science.

[CR15] Nower L, Blaszczynski A (2003). Binge gambling: A neglected concept. International Gambling Studies.

[CR16] Nower L, Blaszczynski A (2008). Recovery in pathological gambling: An imprecise concept. Substance Use and Misuse.

[CR19] Petry NM, Blanco C, Auriacombe M, Borges G, Bucholz K, Crowley TJ, Grant BF, Hasin DS, O’Brien C (2014). An overview of and rationale for changes proposed for pathological gambling in DSM-5. Journal of Gambling Studies.

[CR20] Sklar A, Gupta R, Derevensky J (2010). Binge gambling behaviors reported by youth in a residential drug treatment setting: A qualitative investigation. International Journal of Adolescent Medicine and Health.

[CR22] Spitzer RL, Kroenke K, Williams JB, Löwe B (2006). A brief measure for assessing generalized anxiety disorder: The GAD-7. Archives of Internal Medicine.

[CR23] Viner RM, Taylor B (2007). Adult outcomes of binge drinking in adolescence: Findings from a UK national birth cohort. Journal of Epidemiology and Community Health.

[CR25] Williams RJ, Hann RG, Schopflocher D, West B, McLaughlin P, White N, King K, Flexhaug T (2015). Quinte longitudinal study of gambling and problem gambling.

[CR26] World Health Organization (1992). The ICD-10 classification of mental and behavioural disorders: Clinical descriptions and diagnostic guidelines.

